# Health policymakers’ knowledge and opinions of physicians smoking and tobacco policy control in Lao PDR

**DOI:** 10.1186/1471-2458-12-816

**Published:** 2012-09-21

**Authors:** Vanphanom Sychareun, Alongkone Phengsavanh, Visanou Hansana, Sysavanh Phommachanh, Mayfong Mayxay, Tanja Tomson

**Affiliations:** 1 Faculty of Postgraduate Studies & Research, University of Health Sciences, P.O. Box 7444, Vientiane, Lao PDR; 2 Department of Public Health Sciences, Norrbacka,2nd floor, Karolinska Institutet, Stockholm, Sweden

**Keywords:** Health policymaker, Opinion, Smoking, Medical doctor, Low-income country

## Abstract

**Background:**

In 2007, a regulation on smoke-free health facilities and institutions was adopted by the Lao government. Little is known about health policymakers’ knowledge and opinions regarding tobacco policy control, including physicians’ behaviour. This paper aims to describe the knowledge of Lao health policymakers and their opinions regarding physicians tobacco use and national smoking policy control.

**Methods:**

In 2007, we made a qualitative explorative study with data from a purposive sample of 18 key informants through semi-structured, face-to-face interviews. The key informants, who were heads of departments, directors of hospitals and directors of centres, mainly worked at the national level, and some provincial levels. Content analysis was used.

**Results:**

Policymakers perceived the inadequate implementation of a smoke-free regulation and policy as being a barrier and that the general public may not accept physicians smoking, since they are regarded as role models. Most of the respondents mentioned that regulations or laws related to control of smoking in health institutions are available in Laos, but they lacked detailed knowledge of them probably because regulations as well as the smoke-free policy documents were not widely disseminated. The respondents agreed that anti-smoking education should be integrated in the training curricula, especially in the medical schools, and that the provision of counselling on health consequences from smoking and methods of smoking cessation was important.

**Conclusion:**

This study contributes to tobacco policy evidence and to knowledge regarding factors related to the uptake of evidence into policymaking. Dissemination and implementation of a tobacco control policy nationally, and integration of tobacco cessation training programs in the curricula were found to be productive approaches for improvement.

## Background

The global epidemic of tobacco smoking is expected to impact hardest on low- and middle-income countries (LMICs) [1]. Lao PDR has a high prevalence of smoking among general population as smoking prevalence among adults 15 years and over was 38% (Males- 41%, Females- 15%). The retail price of 20 cigarettes including tax for domestic brands is $0.38-0.76$ [2].

There is a lack of understanding regarding the policy environments within which tobacco control policies are being introduced here. Doctors could play an important role in the identification, assessment, and treatment of smokers [3]. Smoking prevalence among physicians is high in many LMICs. In China, 58% of male and 19% of female physicians were reported as being current cigarette smokers [4]. In 2003, the smoking prevalence among male doctors at a tertiary central hospital in Vientiane, the capital of Laos, was reported at 35% [5]. However, in a recent countrywide survey conducted in 2007, the prevalence of current smoking among Lao doctors was only 9.3% [6].

Doctors are regarded as role models for tobacco control and can be important in the development of a tobacco policy [7]. In contrast, smoking by doctors may inhibit them from counselling patients about smoking and overall tobacco control involvement [8].

In order for an anti-smoking policy to be successful, the relationship between policymakers and practitioners needs to be better understood, as well as the smoking behaviour of the practitioners [9]. Understanding health policymakers’ own opinions towards anti-smoking is essential, since they are considered most important for decisions on health policies, including tobacco control strategy. As part of a study on smoking behaviour and tobacco control among medical doctors in the Lao PDR [6], this study aimed at improved understanding of the opinions of health policymakers regarding smoking among doctors and smoking-free policies at health facilities in order to inform on tobacco control in Lao PDR.

## Methods

### Setting

Lao PDR (Laos) has 17 administrative provinces and one capital city (Vientiane). There are four administrative strata in the health system: central (Ministry, University of Health Sciences, and reference/specialized centers), provincial (provincial health office, provincial and regional hospitals, and auxiliary nursing schools), district (district health offices and district hospitals), and village (health centers) levels. Health facilities encompass four central teaching and referral hospitals, three specialized centres, 16 provincial hospitals, 130 district hospitals, and about 862 health centres [10].

There is no national smoke-free policy, but there are local initiatives e.g. most universities, all Lao Women Union district offices, and some hospitals are smoke-free and a Lao smoke-free local policy was launched by the Ministry of Health in 2007. The same year Ministry of Public Security adopted a smoke-free regulation to ban all workplaces from smoking. A comprehensive tobacco control law was issued in 2009 including prohibition of tobacco advertisement (Article 48) [11].

We carried out in-depth interviews with key informants who were the health policy decision makers within the Lao Government Ministry of Health and public health system.

Data collection for this qualitative descriptive study was conducted in 2007 as part of the country-wide survey on smoking behaviour and tobacco control among medical doctors in Lao PDR [6]. Vientiane Capital and the three largest provinces representing the North (Luangprabang), the Centre (Savannakhet), and the South (Champasack) were purposively chosen for the study.

### Key informants in-depth interviews

The key informants included health policymakers, mostly at the national level, but also a few at provincial levels, all purposively selected based on their position within the Ministry of Health and provincial health departments and hospitals. The national level key informants included the Vice-Minister of Health, responsible for Hygiene and Prevention, Directors or Deputy-Directors, Department of Hygiene and Prevention, and Department of Health Care, Vice-Dean of the Faculty of Medical Sciences, Directors or Vice-Directors of four central teaching hospitals, Director of Vientiane Capital Health Office, and Directors of Centres of Mother and Child Health, the Tuberculosis Center, and the Center of Laboratory and Epidemiology. Two key informants were interviewed in each province, Directors or Deputy-Directors of provincial health and hospital of the three provinces.

### Data collection

Four interviewers with previous medical experience and four note-takers working in pairs, conducted the interviews**.** About 20 key informants were approached, and 18 were recruited and interviewed. If the directors were away at the time of interview, we then contacted the vice directors and interviewed them. Face-to-face in-depth interviews were conducted, using a standardized guideline (Table 1). In addition to taking notes, a tape recorder was used. The interviews took place in the offices of the key informants during official working hours and each interview took between 45 minutes to one hour.

**Table 1:** Interview guide for smoking among health professionals

**Interview guide**
1 Key demographic information (age, general education, administrative positions, working experiences, smoking status)
2 What do you think about smoking among health professionals? Is it acceptable to the general population for medical doctors to smoke? Why? What do you think about the role model of medical doctors in terms of smoking?
3 What makes health professionals smoke?
4 What do you know about the regulation or law related to smoking in the hospitals/teaching faculties?
5 What sort of smoke-free policy is in place at the health facilities/MOH? Is the smoking policy reinforced? If yes, Why? If no, Why?
6 What do you think about providing counselling about the effect of smoking to people? On quitting smoking?
7 Are there any lessons on tobacco control in the training curriculum? What are they?

### Data analysis

All tape-recorded interviews were transcribed by the interviewers and computerized in the Word document format. Content analysis was then used to analyze the data [12,13]. The analysis included a thorough reading of the texts, resulting in the identification of significant statements and phrases, including all those referring to the research questions of extract interest as the themes. The meaning units were identified and then labelled as condensed meaning units. The condensed meanings were labelled with codes, and different sub-categories were tabulated in order to find different and similar sub-themes.

Ethical research approval was obtained from the National Ethical Review Board for Research, Ministry of Health, Vientiane, Lao PDR ref No 132/NECHR. Informed consent was sought and obtained from all individuals participating in face-to-face interviews.

## Results

Table 2 presents the socio-demographic characteristics of the key informants. A total of 18 policy makers (14 males) with a median age of 56 years old (min = 42 and max = 68) and working years of 21.5 (min = 10 and max = 34), respectively, were included. All except one were medical doctors and five of them had post-graduate training. Six were current smokers, five ex-smokers, and seven had never smoked. Four themes emerged from the content analysis. The first explores “Perspectives of smoking among medical doctors”. The second described “Smoke-free policy or law related to smoking at the health institutions”. The third describes “Factors influencing smoking practice”. The fourth includes “Provision of prevention of tobacco control” (Table 3).

**Table 2:** Socio-demographic characteristics

	**N**	**Percentage**
Age	56 (Min = 42, Max = 68 )	
Sex		
Male	14	50.0
Female	14	50.0
Working experiences	21.5 (Min = 10, Max = 34)	
Qualification		
Medical Doctor	11	61.1
Master	4	22.2
Specialist	2	11.1
PhD	1	5.5
Smoking status		
Currently smoking	6	33.3
Never smoker	5	27.8
Ex smoker	7	38.9

**Table 3:** Example of analysis of codes, categories and themes

**Codes**	**Categories**	**Themes**
Knowledgeable of the health effects of smoking	Unacceptable/acceptable for among medical doctors to smoke	Policy maker’s perspectives of smoking among medical doctors
Socializing		
Individual behaviour		
Non smoking	Role model of medical doctors on smoking	
Implementing policy		
Quit smoking		
No awareness of smoke-free regulation policy	Limited awareness on smoke-free policy	Smoking-free policy/law at the health institutions
No wide dissemination of smoking free policy		
Smoking prohibition/	Availability of smoking-free policy or law	
No smoking prohibition		
Smoking areas		
Peer pressure	Peer influence	Factors influencing smoking practice
Manhood		
Socialization		
Overload	Working conditions	
Night duty		
Stress		
MD as trusted source	Perspective on the counselling of smoking	Provision of tobacco control
Knowledgeable & Skilful		
Not ready to be a counselor		
Radio, leaflet, posters	Perspective on counselling method	
Face-to-face counselling		
Hotline counseling		
Combination methods		
-No existing lessons/ No official lessons	Integration of lessons on anti-smoking in the training curriculum	
-Political and economic reasons		
-Limitation and inadequate smoke-free policy regulation		

### Theme 1: Policy maker’s perspective of smoking among medical doctors

#### Unacceptable/acceptable for medical doctors to smoke

The majority of the respondents (15/18) agreed that the general public might not accept physicians smoking since medical doctors are regarded as societal role models. In addition, the doctors know more about the health effects of smoking than the general public.

"“In my opinion, people who do not smoke would not accept physicians smoking; they would criticize the physicians smoking because they may think that health care providers are knowledgeable persons and should not smoke and have to be good role models” (Male, 57 years old, never smoker)"

A small number (3/18) of the male health policymakers realized that the general public may accept doctors’ smoking behaviour, which is seen as a common practice when the doctors socialize. Furthermore, the policymakers perceived smoking as being an individual behaviour which does not affect their skill of providing services to the patients. So, respecting medical doctors is not dependent on their smoking status but rather on their morals, knowledge and clinical practices. One key informant mentioned:

"“Medical care provision is not based on the health providers’ smoking, but on their skills and abilities to provide health care” (Male, 52 years old, ex-smoker)."

#### Role model of medical doctors on smoking

The majority of the policymakers (16/18) perceived that health professionals should be role models for smoking cessation and a smoke-free life in order to implement smoking-free policies in their health facilities.

Being a good role model would be useful for them to persuade and convince the general public and patients to stay away from the hazardous practice.

"“The medical doctors, including policymakers should begin to be good role model of non-smoking in order to increase people’s health beliefs in the hazards of smoking. Otherwise, people will not trust them whenever they provide them counselling on the health consequences of smoking” (Male, 67 years old, never smoker)."

### Theme 2: Smoke-free policy at the health institutions

#### Limited awareness on smoke-free policy/Ministry of Health

Most respondents (14/18) said they were not aware of the smoke-free zone within MOH and an existing restraint regarding the implementation of a smoke-free zone. Some said they did not know about the MOH smoke-free zone regulation. Few respondents (4/18) were able to specify any health policy details related to smoking, and most of them said there was no officially implemented smoke-free policy in their workplace.

"“Some physicians were not aware of smoking policy, because they did not receive any information related to smoking policy” (Female, 53 years old, never smoker)."

#### Availability of smoke-free regulation or law at the health institutions or facilities

Most respondents (14/18) said that a current regulation or law related to smoking in a health institution was now in effect. Some health professionals seemed to have created their own regulation about prohibiting smoking at the hospitals. For example, smoking is prohibited in the hospital areas for all health staff as well as patients and their relatives.

"“Recently, hospitals prohibited smoking by putting up “no smoking” signs in waiting rooms and patient wards” (Female, 45 years, never smoker)"

In contrast, few (4/18) of them mentioned having heard anything about the smoke-free policy from the central level, and so they did not have any regulations or laws related to smoking in their health care facilities in place.

"“I did not hear about any smoke-free policy at the health facilities. Perhaps, the smoke-free policy was implemented at the central level and was not disseminated to the provincial levels” (Male, 57 years, smoker)"

### Theme 3: Factors influencing smoking behaviour

#### Influences on the smoking practices of medical doctors

"“Smoking is a social behaviour, which is also influenced by peers, working conditions and which most people started after working in the health sector”. Director of Hospital (male, 58 years)"

#### Peer influence

Some doctors were occasional smokers, who might experience peer pressure, when they were in a group drinking alcohol. This seems to indicate that they try to adapt or become accustomed to the smoking behaviour in order to increase their social status.

"“I only smoked when I went out with my friends or my colleagues, especially when we went out to drink at the beer shop”. (Male, 45 years old, smoker)"

#### Working conditions

Some smokers stated that physicians have difficulties in handling work-related stress, especially when on round-the-clock night duty at the hospitals. Several of the smokers said that they smoked more often when on duty with long working hours, and lack of sleep.

"“I smoked a lot when I worked hard, especially when I was on night duty. For example, when there were a lot of new admissions during the night, I used to smoke a lot” (Male, 55 years old, smoker)."

### Theme 4: Provision of tobacco control

#### Opinions about counselling on smoking consequences and smoking cessation

Most policymakers (16/18) agreed that counselling people on smoking effects is good because people will quit smoking. Most medical doctors did not have any experience of providing health counselling about the effects of tobacco and how to quit smoking; usually they talked in general terms about the health effect of smoking.

"“Physicians are not ready to provide counselling on how to quit smoking because they also smoke and patients do not trust them. The other issue is that they are not ready to provide counselling because they have not been trained properly and they have never provided counselling to patients before” (Female, 45 years old, non smoker)."

#### Method of providing health education on smoking

The respondents´ opinions of the health education procedure can be classified in different ways. First, health education should be generated in hospitals through radio and posters about health impacts from smoking. Secondly, face-to-face counselling can be applied at the end of a consultation capitalizing on the interaction between counsellors (doctor or nurse) and patients. Thirdly, a stop smoking hotline for a one-on-one counselling over the phone, as a free resource that can be tapped into anytime may increase their chances of quitting.

"“There are many ways of providing health education on smoking prevention or cessation to patients and the general public such as posters, leaflets, radio and medical consultation to patients” (Male, 45 years old, smoker)."

"“In addition a hotline counselling centre should be established in order to provide counselling for those not having the chance to visit a medical health care centre or hospital but which could still offer smokers privacy and confidentiality” (Male, aged 55 years old, non-smoker)."

#### Integration of lessons on tobacco control in the training curriculum

The majority of the respondents (13/18) said that so far lessons on tobacco control have not been integrated into any training curricula due to political and economic issues related to tobacco production and national economy. Most of them added that as long as the MOH implementation of a smoke-free policy remains limited and inadequate, the idea of integration might not be possible.

"“It is not meaningful or useful to integrate smoking issues into medical curricula, when tobacco is being produced, maybe even locally, and imported, and there are limitations in smoking-related MOH policies and regulations. So these ideas are in conflict with one another. For instance, giving lessons on the impact of smoking might not work, while tobacco production remains legal and related to the national economy.” (Female, 55 years old, non -smoker)."

The medical curricula did not contain any specific training on tobacco and smoking issues. The training given was a part of the non-communicable disease program, covering such topics as risk factors for lung cancer, hypertension and so on. The most popular method was when the topic was discussed during the teaching of other subjects. The topic areas were mainly related to knowledge about tobacco.

"“There was no training in the medical curriculum at medical school, it was just integrated into other subjects. The medical students did not learn about smoking cessation, they just learned about the health effects of smoking” (Male, age 65 years old)."

## Discussion

The Lao health policymakers were positive towards having national tobacco control. Yet, although tobacco control regulations and laws were said to be in operation at health institutions or facilities, policymakers knew little about the content of these smoke-free regulations, because the information had been poorly disseminated. They emphasized the importance of including tobacco control training, including counselling, in the curricula. They also stated that since physicians and other health professionals can play an important role in assisting smokers in quitting smoking, they must be good role models for a smoke-free lifestyle [14-18].

Informants agreed that physicians who smoke may not be accepted by the public and that this can generate a negative image of health professionals [8]. Here, it is noteworthy that one third of them were themselves smokers and another third ex-smokers. In Lao society, physicians are generally the key people who provide counselling on how to stop smoking, and therefore they should be a role model for smoking cessation. However, in practice not all doctors who provide cessation advice are non-smokers. In China, a majority of smoking physicians stated they provided smoking cessation counselling [4]. The smoking physicians were less likely to discourage patients from smoking or to provide smoking cessation counselling when compared to those who did not smoke [4,19]. One of the reasons that physicians who themselves are smokers offer less clinical cessation activities may be that they lack confidence in their ability to counsel patients [20] as mentioned by the health policymakers in our study. Previous studies have suggested that medical doctors are perceived as societal leaders in matters related to health, and that they accept this role as part of their profession [21].

Most of the responders had limited knowledge regarding existing regulations and how these were implemented. Once they become aware of the regulations, the Lao health policymakers seem strongly committed to the implementation of a nation-wide policy. This is consistent with findings in another study of a national tobacco policy in Lao PDR, but some smoking doctors maintained a negative attitude toward a smoke-free policy [22]. A regulation on non-smoking in health facilities has been issued and implemented in some central hospitals in Vientiane, but not on a national scale. There is strong evidence that higher cigarette taxes, advertising bans, clean air laws and media campaigns can reduce adult smoking rates, especially when combined in a comprehensive overall strategy [9].

Policymakers perceived that medical doctors lacked counseling skills. Previous studies suggested that physicians have a crucial role in advising and helping smoking patients to stop [23]**,** but lacked the tools [24].

The new generation of medical students, should also acquire these basic knowledge and skills. Nowadays, international organizations urge educators to include information on tobacco in the undergraduate curricula of future health professionals [25]. Relevant course materials need to be integrated into the medical school curriculum [26]. Lancaster et al. (2000) [27] concluded that health care providers, who received smoking cessation training, were significantly more likely to intervene with patients who use tobacco than those who were not formally trained. Thus training opens opportunities to promote cessation and shift professional and societal norms away from tobacco use [23].

Although doctors and other health professionals can play a vital role in tobacco control, they alone will not be able to accomplish this huge task. In order to successfully implement a national tobacco control policy in Laos, there is a need for coordination and cooperation among multiple stakeholders [18,28].

This qualitative study included 18 key informants, policy makers from central and provincial hospitals and from University and Ministry of Health (MOH). Only two of the approached refused to participate due to their busy time and they were not available even though the research team made appointment with them twice. Those not participating were the directors from center for malaria, parasitology, entomology and center for control of HIV/AIDS/STIs. The strength of qualitative research is its ability to provide complex textual descriptions of how people experience a given research issue. Study design is iterative, that is, data collection and research questions are adjusted according to what is learned and not to the number of interviewees.

## Conclusions

This study contributes to tobacco policy evidence and also to our knowledge regarding factors related to the uptake of evidence into policy making [29].

The policymakers were aware of the anti-smoking and tobacco control laws but implementation was not enforced. Lao policymakers’ support for integration of anti-smoking lessons in the training curricula is a good start of tobacco control. The focus of future studies could be to find out if and how the new tobacco law is implemented.

## Competing interests

The authors declare that they have no competing interests.

## Authors’ contributions

VS designed the project, developed the question guide, analysed the data and wrote the first draft of the manuscript. AP and VH participated in the design of the research project and carried out data collection. MM and SP did preliminary data analysis and commented on the draft manuscript. TT participated in the design of the study, commented on and revised the manuscript. All authors have read and agreed on the final version of this manuscript.

## Pre-publication history

The pre-publication history for this paper can be accessed here:

http://www.biomedcentral.com/1471-2458/12/816/prepub
